# Three-dimensional computed tomography angiography for the preoperative evaluation of coronary artery disease in lung cancer patients

**DOI:** 10.1186/1477-7819-11-164

**Published:** 2013-07-20

**Authors:** Fumiaki Watanabe, Osamu Hataji, Kentaro Ito, Corina N D’Alessandro-Gabazza, Masahiro Naito, Hideo Morooka, Esteban C Gabazza, Yukio Mizutani, Maki Ohi, Motoshi Takao, Hideto Shimpo, Isao Yada

**Affiliations:** 1 Respiratory Center, Matsusaka Municipal Hospital, Tonomachi 1550, Matsusaka City, Mie 515-8544 Japan; 2 Department of Radiology, Matsusaka Municipal Hospital, Tonomachi 1550, Matsusaka City, Mie 515-8544 Japan; 3 Department of Cardiovascular Medicine, Matsusaka Municipal Hospital, Tonomachi 1550, Matsusaka City, Mie 515-8544 Japan; 4 Department of Immunology, Mie University Faculty and Graduate School of Medicine, Edobashi 2-174Mie 514-8507 Tsu City, Japan; 5 Department of General Thoracic and Cardiovascular Surgery, Mie University School of Medicine, 2-174 EdobashiMie 514-8507 Tsu City, Japan

**Keywords:** Aging, Coronary ischemic disease, Lung cancer, Surgery

## Abstract

**Background:**

The number of elderly patients undergoing surgery for lung cancer is increasing. In this study, we assessed the usefulness of three-dimensional computed tomographicangiography (3D-CTA) for the detection of coronary disease in the elderly before surgical intervention for lung cancer.

**Methods:**

One hundred twenty patients admitted to our institution for lung cancer resection were enrolled in the study. 3D-CTA was performed in all 120 patients.

**Results:**

Seventy-one patients had normal findings, and forty-nine patients showed coronary stenosis on 3D-CTA examination. Among the latter 49 patients, 24 with slight stenosis underwent lung tumor resection, 23 had coronary angiography for severe stenosis before lung surgery and 2 were not eligible for lung resection because of very severe coronary stenosis. The diagnostic value of 3D-CTA was better than conventional CT.

**Conclusions:**

This study suggests the usefulness of 3D-CTA for the preoperative diagnosis of coronary ischemic disease in elderly lung cancer patients.

## Background

The aging population is increasing in developed countries, including the United States, Canada and Australia as well as European countries, and the increase is even greater in Japan [1-3]. Although prolonged life expectancy is one of the biggest achievements of humankind, expansion of the oldest population also implies a rise in age-related diseases, including malignancies and cardiovascular disorders [1]. Among malignant diseases in the aging population, lung cancer is the leading cause of death in Japan and worldwide [4-6]. Patients with lung cancer have a poor prognosis, with only 15% of them being eligible for surgical resection [4,6]. Although the therapeutic response is limited, patients with advanced disease are usually treated with systemic chemotherapy or tyrosine inhibitors [4,6].

In Japan, surgery is not usually indicated as a therapeutic option for the oldest group of lung cancer patients (older than 80years of age) because of poor performance status [7,8]. Because the performance status of some elderly groups with lung cancer has dramatically improved, aging is no longer an exclusion criterion for surgical intervention. However, the presence of concomitant diseases, such as emphysema, high blood arterial hypertension, coronary ischemic disease, cerebrovascular disorders and diabetes mellitus, are not uncommon in the elderly. Therefore, a meticulous presurgical examination is critical to avoiding complications during and after surgery [9-11]. In particular, the risk of complications is much higher in the elderly people with coronary disease associated with diabetes mellitus or the metabolic syndrome [12].

In the present study, we evaluated the usefulness of three-dimensional computed tomographicangiography (3D-CTA) for the detection of coronary disease in the elderly before surgical intervention for lung cancer.

## Methods

One hundred twenty patients (69 males, and51 females; mean age, 71.4±9.0 years old) admitted to our institution from 1 November 2009 through 30 September 2012 for surgical intervention of lung tumors were enrolled in the study. Among all patients, 111 had primary lung cancer, 2 had atypical adenomatous hyperplasia and 7 had metastatic lung tumors spread from other distal organ primary tumors. The histological type of the lung primary tumors was as follows: 86 adenocarcinomas, 19 epidermoid carcinomas, 1 large-cell carcinoma, 3 small cell carcinomas and 2 multiple lung cancers. The lung cancer stages were as follows: IA, 71 patients; IB, 20; IIA, 8; IIB, 2; IIIA, 4; IIIb, 2; and IV, 4 patients. Other underlying diseases of the patients and smoking history are described in Table 1.

**Table 1:** **Characteristics of the patients with normal and abnormal 3D-CTA findings**^a^

	**Patients with normal 3D-CTA findings (*n*=71)**		**Patients with abnormal 3D-CTA findings (*n*=49)**	
	**Number**	**(%)**	**Number**	**(%)**
Sex				
Male	36	(50.7)	33	(67.3)
Female	35	(49.3)	16	(32.7)
Age				
>75years old	26	(36.6)	27	(55.1)
Smoking (+)	31	(43.7)	33	(67.3)
Underlying disorders				
Diabetes mellitus	5	(7.0)	11	(22.4)
Arterial hypertension	26	(36.6)	26	(53.1)
Hyperlipidemia	15	(21.1)	9	(18.4)

^a^ 3D-CTA, three-dimensional computed tomographicangiography.

A high-resolution CT scanner, the Philips Brilliance iCT (Philips Healthcare, Best, The Netherlands) with both 128- and 256-slice configurations, was used. The scanning parameters were as follows: 0.625mm collimation×128, pitch 0.14, rotation time 0.27s, thickness 0.8mm and slice increment 0.4mm. The Nemoto Dual Shot contrast injector (NemotoKyorindo Co, Ltd, Tokyo, Japan) was used for contrast administration. An electrocardiography gated volume scan was performed to assess coronary and pulmonary arteries at the same time. The whole lungs were scanned and photographed after a breath hold. The timing to start scanning was determined by using the bolus tracking method. The ascending aorta was taken as the region of interest, and the scan imaging was started when the CT values reached 100. CT data were transferred to an imaging analysis system (Extended Brilliance Workspace; Philips Healthcare) for image reconstruction.

The presence of calcification visualized by conventional CT was evaluated and classified as grade 0, no calcification; grade 1, calcification in one arterial branch or diffuse calcification; grade 2, calcification in two arterial branches; and grade 3, calcification in three or more branches. Written informed consent was obtained from each patient before the beginning of the study.

## Results

Preoperative examination with 3D-CTA was performed in 120 patients. Of these, 71 showed normal findings and 49 (40.8%) showed coronary stenosis. The grade of coronary stenosis in these 49 patients was slight in 24 patients and severe in 25 patients. Patients (*n*=24; 48.9%) with a slight grade of coronary stenosis based on 3D-CTA findings underwent lung tumor resection. Among the patients with severe coronary stenosis, 23 (46.9%) underwent coronary angiography before lung surgery and 2 (4.2%) were considered ineligible for lung resection.

Among the 23 patients who under went coronary angiography, percutaneous coronary intervention (PCI) (diagnostic yield 69.5%) was indicated in 16 patients, and in 7 patients this intervention was considered unnecessary. Of 16 patients with indications for PCI, 1 had surgery after placement of a bare metal stent and the remaining 15 underwent lung resection while receiving continuous infusion of a vasodilator (nicorandil). PCI was scheduled in the latter 15 patients in a watch-and-wait fashion. In our hospital, stent implantation is performed in all angioplasty interventions. The coronary arteries that were surgically treated in 16 cases were as follows: anterior descending branch (8 cases), circumflex branch (4 cases), right coronary artery (1 case), anterior descending branch plus diagonal branch (1 case), anterior descending branch plus right coronary artery (1 case)and anterior descending branch plus right coronary artery plus circumflex branch (1 case).

**Figure 1:**
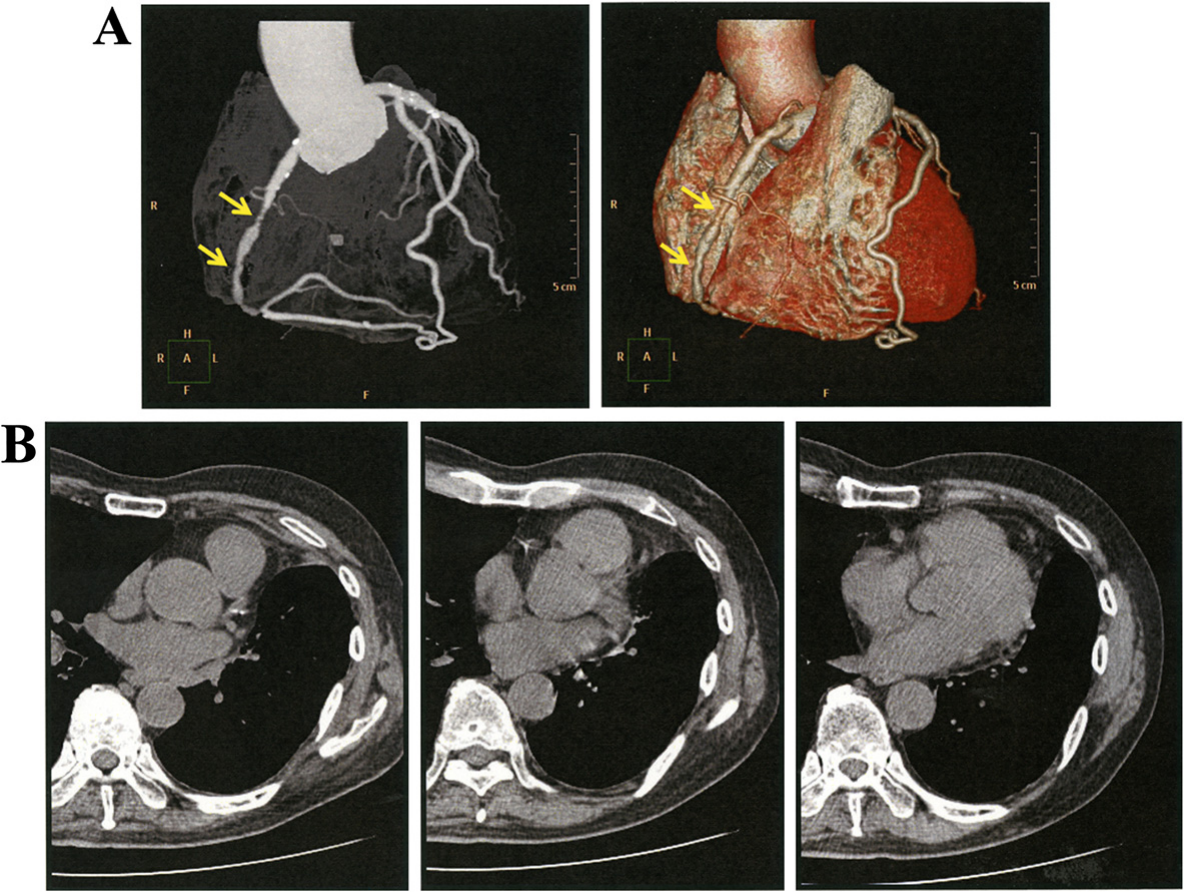
**3D-CTA and conventional CT findings.** Multiple areas with coronary stenosis (arrows) visualized by 3D-CTA **(A)** in an 82-year-old male patient with calcification grade 1 diagnosed by conventional CT **(B)**.

Conventional CT showed only findings of calcification grade 0 or1 in 11 patients judged as having severe coronary stenosis on the basis of3D-CTA and in 7 patients who actually required PCI (Table 2 and Figure 1). Furthermore, coronary angiography was not indicated in three patients with calcification grade 3 ascertained by conventional CT, because the 3D-CTA study showed no stenosis. The need of PCI was not possible to judge solely on the basis of conventional CT findings.

**Table 2:** **Relationship between 3D-CTA and conventional CT grading**^a^

**3D-CTA**					
**Conventional**	**Normal**	**Slight**	**Severe**	**PCI**	**Ineligible for lung resection**
**CT grading**	**(*n*=71)**	**(*n*=24)**	**(*n*=25)**	**(*n*=16)**	**(*n*=2)**
Grade 0	46	7	4	3	0
Grade 1	17	11	7	4	0
Grade 2	5	5	8	4	1
Grade 3	3	1	6	5	1

^a^ 3D-CTA, three-dimensional computed tomographic angiography; normal grade, no calcification; slight grade, calcification in 1 vessel; moderate grade, calcification in two vessels; severe grade, calcification in three or more vessels; PCI, percutaneous coronary intervention.

## Discussion

In this study, we have shown the usefulness of 3D-CTA for the detection of asymptomatic coronary ischemic disease in elderly patients before surgical resection of lung tumors. In an attempt to prevent coronary events during the perioperative period, several guidelines for perioperative cardiovascular assessment have been estab-lished before patients undergo noncardiac surgery [13-15]. Owing to the poor positive predictive value of noninvasive cardiac stress tests for perioperative evaluation, it is often necessary in some patients to use alternative but invasive procedures to make decisions about whether to perform preoperative coronary revascularization before elective noncardiac surgery [16]. The perioperative risk of coronary artery disease, including myocardial infarction, cardiac arrhythmia and cardiac arrest, is high in patients undergoing surgery. The precise mechanism of myocardial ischemic events during the perioperative period is unclear, but surgical stress-associated myocardial ischemia and/or rupture of coronary atherosclerotic plaque are believed to play critical roles [17,18].

The recent advances in the technology of multislice CT have expanded the clinical application of CT scanning as a tool for the diagnosis of several diseases, including cardiovascular disorders [19-22]. Furthermore, analysis using imaging system software has allowed the three-dimensional reconstruction of vascular branches by providing images with quality even better than images obtained using conventional angiography [21,22]. In the present study, we used 3D-CTA for preoperative evaluation of coronary ischemic disease in agroup of elderly lung cancer patients with surgical indications. 3D-CTA showed the presence of coronary stenosis in almost half of the cases, and many of them required PCI. Surgical procedures in subjects with coronary atherosclerotic disease are associated with the risk of morbidity and mortality in the perioperative period [17,23]. Therefore, the results of the present study strengthen the value of 3D-CTA for the diagnosis of coronary ischemic disease and support its use for the preoperative evaluation of elderly lung cancer patients.

One limitation of our present study is that coronary angiography was not performed in all patients; consequently, the number of false-negatives using this 3D-CTA diagnostic method is unclear. However, none of our patients found to have no coronary stenosis by 3D-CTA in the preoperative period had vascular complications after surgery, further supporting the usefulness of 3D-CTA as a screening tool for coronary disease in elderly patients before performing surgery for lung tumors.

## Conclusions

In brief, the present study suggests the usefulness of 3D-CTA for the preoperative diagnosis of coronary ischemic disease in elderly lung cancer patients.

### Consent

Written informed consent was obtained from the patient for the publication of this report and any accompanying images.

## Competing interest

None of the authors had any competing interests regarding the results reported in this study.

## Authors’ contributions

FW participated in the design of the present study and prepared the first draft of the manuscript. OH, CNDG, KI, MN, MO and HM participated in data collection and analysis. YM, MT, HS and ECG participated in the acquisition of the patients’ medical records. IY participated in the acquisition of medical records and coordinated the whole study. All authors read and approved the final manuscript.
